# Combined Techniques of Non-invasive ^99m^Tc-Besilesomab/^99m^Tc-Sulfur Colloid with Hybrid SPECT/CT Imaging in Characterising Cellulitis from Symptomatic Perimegaprosthetic Infection: A Case Report

**DOI:** 10.5704/MOJ.2011.032

**Published:** 2020-11

**Authors:** N Tagiling, MF Mohd-Rohani, WF Wan-Sohaimi, WI Faisham, NM Nawi

**Affiliations:** 1 Department of Nuclear Medicine, Radiotherapy and Oncology, Universiti Sains Malaysia, Kubang Kerian, Malaysia; 2 Nuclear Medicine Department, Hospital Kuala Lumpur, Kuala Lumpur, Malaysia; 3 Department of Orthopaedics, Universiti Sains Malaysia, Kubang Kerian, Malaysia

**Keywords:** megaprosthesis, periprosthetic infection, nuclear medicine, single-photon emission computed tomography/computed tomography, radiopharmaceuticals

## Abstract

Megaprosthesis is used to restore the form and function of massive skeletal defects, but it is accompanied by risks of failure, mainly due to perimegaprosthetic infection (PMI). In practice, the diagnosis of infected megaprosthesis among patients with a high index of clinical suspicion, elevated serological markers, and multiple negative or inconclusive imaging can be very challenging and poses a diagnostic conundrum to many orthopaedic surgeons. We present the case of a symptomatic 26-year-old female with large B-cell lymphoma who developed cellulitis with suspected complication of PMI 15 months post-implantation. The combination of advanced nuclear medicine imaging strategies, i.e., ^99m^Tc-besilesomab/^99m^Tc-sulfur colloid scintigraphy with hybrid single-photon emission computed tomography/computed tomography (SPECT/CT) scanning helps to characterise and delineate both infections. Invasive procedures such as joint aspiration and biopsy were avoided, and the patient was successfully treated with antibiotics. Hence, we report a case where advanced imaging modalities were decisive in the investigation of PMI.

## Introduction

Megaprosthesis reconstruction is an established orthopaedic procedure of restoring large bone defects for the treatment of both neoplastic and non-neoplastic conditions. However, it is often associated with a high risk of complication^1^. Perimegaprosthetic infection (PMI) is one of the most daunting complications facing orthopaedic surgeons due to the complexity of its management. Statistics of PMI were found to be highly variable, ranging from 3% to > 30%^1^.

In a recently-published 2019 consensus guideline for prosthetic joint infection diagnosis, the working delegates have unanimously recommended the use of advanced nuclear medicine (NM) imaging to visualise functional changes surrounding skeletal structures upon persisting suspicion of infection^2^. Newer technology such as hybrid single-photon emission computed tomography/computed tomography (SPECT/CT) scanner was also mentioned in the consensus statement, stating its prospective superiority compared to conventional planar imaging in localising potentially infected sites. Despite that, there is still a limited amount of literature regarding its use, and establishing a reliable diagnosis can be more challenging for megaprosthesis than standard joint replacement^1,2^.

In this report, we demonstrate the utility of advanced NM imaging, together with hybrid SPECT/CT for the investigation of PMI.

## Case Report

**Fig. 1:**
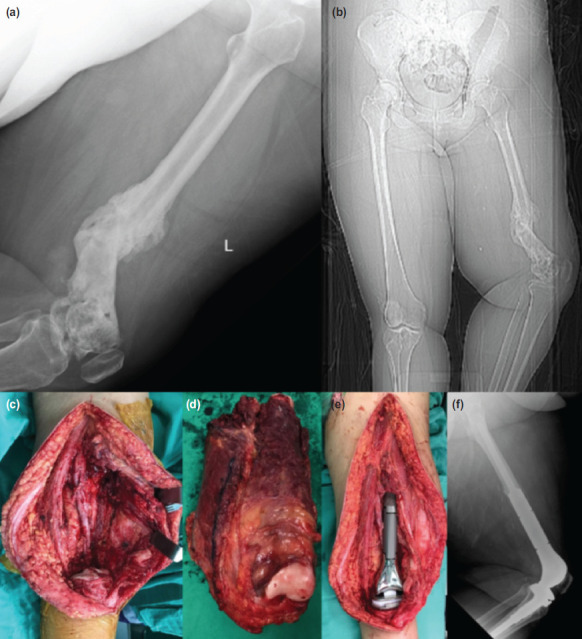
(a,b) Radiograph of left femur deformity with callus formation and angulation of distal fragment anterolaterally in keeping with malunion, following chemotherapy and prolonged antibiotic. (c,d,e) Wide resection margin of bone and surrounding soft tissue to achieve good local control for megaprosthesis reconstruction. (f) Post-operation radiograph of distal left femur shows megaprosthesis in situ.

A 26-year-old female with underlying primary left inguinal diffuse large B-cell lymphoma (DLBCL) who had completed six cycles of chemotherapy presented with a recurrent left knee swelling 1-year post-chemotherapy. An ^18^F-fluorodeoxyglucose (^18^F-FDG) positron emission tomography/computed tomography (PET/CT) study was performed to rule out lymphoma recurrence. She was concluded to have active lymphomatous disease at the distal part of the left femur and complicated with a pathological fracture. Bone biopsy confirmed the presence of DLBCL; however, the wound was complicated with chronic infection with discharge. Systemic chemotherapy and prolonged antibiotic treatment were able to control disease progression and control the infection. The pathological fracture united with deformity (Fig. 1a and b), knee stiffness in 30º flexion deformity, and 5cm shortening. Wide resection of the affected left distal femur was then performed to achieve local control for both malignancy and chronic infection (Fig. 1c and d). The segment defect was reconstructed with a modular megaprosthesis (Fig. 1e and f). However, histopathological examination later revealed features of chronic osteomyelitis and negative for malignancy.

Pre-operatively, her white cell count (WCC) and erythrocyte sedimentation rate (ESR) levels were slightly raised. Subsequently, her post-operative WCC increased nearly 3-folds. She was then treated for osteomyelitis, and her WCC levels improved to normal range within two weeks. Follow-ups in the ensuing months after surgery were uneventful, and there were no signs and symptoms of surgical site infection, implant failure, or neurovascular compromise.

Fifteen months later, she presented to the hospital with a week-long fever with chills and rigors, and debilitating pain at the left inguinal region. There was a swelling in the left inguinal region measuring 4.0cm × 3.0cm, firm in consistency, smooth, mobile, and non-tender on palpation. Examination of the left lower limb revealed swelling up to below the knee with multiple tender erythematous areas over the medial aspect of the thigh but with no blister, discharge, or crepitation. The surgical scar at the medial aspect of the left knee was well healed. Blood investigation revealed markedly elevated levels of inflammatory markers: WCC = 31.6 × 109/L, C-reactive protein (CRP) = 193 mg/L, and ESR = 84mm/hour. The initial impressions for her were either recurrence of lymphoma, cellulitis, PMI, and deep venous thrombosis (DVT). Plain radiographs showed no evidence of implant loosening in the left knee, while ultrasonography of the left lower limb was negative for DVT. Blood cultures showed no growth of microorganisms. She was then treated for cellulitis with intravenous Ceftriaxone and oral Doxycycline. The antibiotics regime was later upgraded to intravenous Vancomycin, followed by intravenous Ceftaroline due to fluctuating fever. Given her fever pattern and persistently elevated inflammatory markers, cellulitis of the left knee region with the possibility of an infected prosthetic implant needs to be considered.

**Fig. 2:**
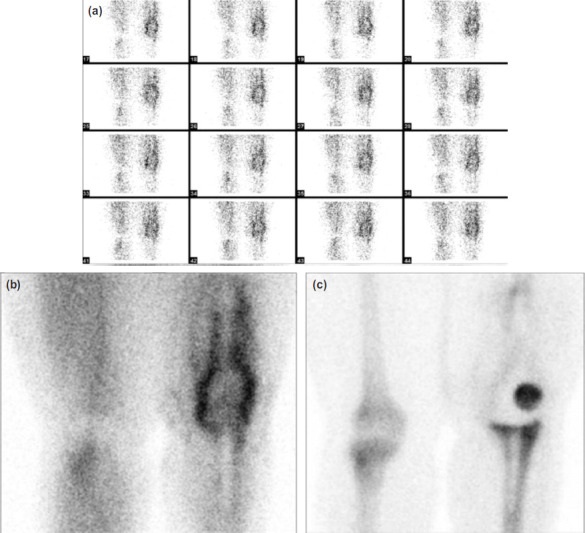
(a) Flow phase of bone scan showed increased perfusion in the left knee joint. (b) Anterior view of knee region for blood pool phase showed increase in radiotracer uptake at the periphery of prosthesis at distal left femur, (c) while delayed phase revealed radiotracer uptake at the left patella bone, and medial and lateral aspect of left proximal tibial prosthesis.

A three-phase bone single-photon emission computed tomography (SPECT) scan with technetium-99m methylene diphosphonate (^99m^Tc-MDP) radiotracer was then ordered and showed increased uptake in the left knee region for both the blood flow (Fig. 2a) and blood pool phases (Fig. 2b) – indicative of soft tissue inflammatory or infective process. The two hours delayed phase bone scan (Fig. 2c) showed no abnormal tracer accumulation (negative delayed phase) in the left distal femoral prosthesis, which is suggestive of cellulitis. However, an increase in radiotracer uptake was demonstrated at the medial and lateral aspect of the left proximal tibial prosthesis, which could either be from prosthetic infection or bone remodeling.

**Fig. 3:**
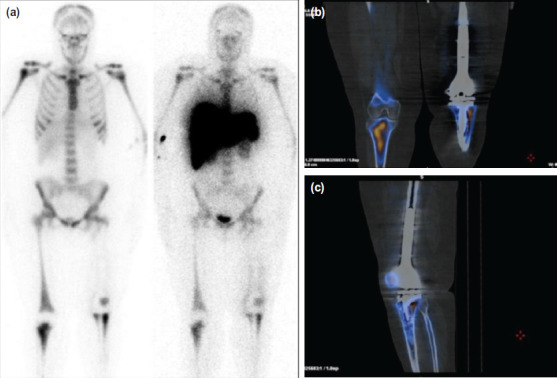
(a) Anterior view of whole-body scan with ^99m^Tc-besilesomab (left) and ^99m^Tc-sulfur colloid (right). Both images showed concordant radiotracer uptake at the left patella, and medial and lateral aspect of left proximal tibia prosthesis. No abnormal radiotracer accumulation was seen in the distal left femoral prosthesis, and tracer distribution elsewhere is physiological. Hybrid SPECT/CT image for ^99m^Tc-besilesomab in (b) coronal and (c) sagittal view. The addition of CT helps to delineate and localize the physiologic uptake pattern of bone marrow. No abnormal uptake were observed on the lower limb soft tissue.

She was then subjected to two NM imaging studies with anti-granulocyte monoclonal antibody scintigraphy with CT (^99m^Tc-besilesomab SPECT/CT) and bone marrow scintigraphy (^99m^Tc-sulfur colloid SPECT) on two separate days. Both of the imaging procedures demonstrated congruent radiotracer uptake at the left patella and the medial and lateral aspect of the left proximal tibial (Fig. 3a). Plus, the hybrid SPECT/CT images for ^99m^Tc-besilesomab showed correct localisation of physiological marrow uptake at the proximal tibia, and there is no abnormal uptake found within the soft tissue region across the anatomical planes (Fig. 3b and c). Hence, the diagnosis of PMI was excluded and obviates the need for implant removal.

The patient continued to be treated for cellulitis with intravenous antibiotics and subsequently showed clinical improvement, as evidenced by her fever resolution and reduction of her left inguinal and left lower limb swelling. Both of her WCC and CRP levels showed marked improvement to 7.8 x 109/L and 24mg/L, respectively. Post-discharge, she showed no signs or symptoms of infection at her 12-month follow-up in the clinic and was able to ambulate well without assistance.

## Discussion

The typical diagnostic algorithm for prosthetic infection comprised of a combination of clinical findings, serological tests, joint aspiration, microbiology, histological evaluation of periprosthetic tissue, and intra-operative inspection^3^. It is worth noting that up until today, there is no single routine test available that can diagnose infected prostheses with sufficient accuracy^2^. For instance, CRP and ESR levels may be elevated without an identifiable cause, while joint aspiration may be dry or inconclusive in low-grade infection. Besides, biopsies are often not advocated as it is an invasive technique. Non-invasive modalities in radiology may support the diagnosis, but their role is non-definitive^3^. Plain radiography is usually performed as part of the initial diagnostic workup to identify non-infectious causes of the symptoms, i.e., periprosthetic fracture or dislocation. The visualisation of lucencies or loosening of the prosthesis may suggest infection, but it is neither sensitive nor specific. Cross-sectional techniques such as standard CT-scan and magnetic resonance imaging can also be beneficial but are hindered by metallic hardware artifacts.

Unlike conventional imaging, NM imaging uses specific radioactive pharmaceuticals or radiotracers to visualise and detect orthopaedic infections^4^. A three-phase bone scan is usually performed with ^99m^Tc-labeled diphosphonates as a first-line screening method with a negative scan ruling out prosthetic infection with a high degree of certainty. Yet, it is unreliable as the diagnostic accuracy for suspected infections within the first two- to five-year period is low^2^. The findings from the bone scan may demonstrate an increase in uptake after surgery due to bone remodeling, which can lead to false-positive interpretation. In our case, the patient presented less than two years after her megaprosthesis implantation.

It was reported that patients who develop cellulitis are at risk of developing secondary prosthetic infection^5^. The risk is even higher among immunocompromised patients, such as those receiving chemotherapy for cancer treatment. In terms of location, infection of the proximal tibia is more common than other sites due to the reconstruction of the patellar tendon, limited soft tissue, and its linkage to the knee. This corroborated our suspicion for PMI, as observed by the increased uptake of ^99m^Tc-MDP at the proximal tibial during the delayed-phase bone scan.

The combined anti-granulocyte SPECT/CT and bone marrow SPECT was ensued to increase the diagnostic accuracy and confidence in differentiating infection from post-surgical changes. Besilesomab is an anti-granulocyte antibody kit that utilises murine immunoglobulin. It accumulates in the bone marrow, and at the same time, attach to a non-specific cross-reacting antigen (NCA-95) that is present in the cytoplasm and on the cell membranes of granulocytes. Upon injection with the ^99m^Tc-besilesomab, the monoclonal antibody carries the radioactivity to the target antigen. As granulocytes are abundant at the site of infection, the ^99m^Tc-besilesomab will show intense uptake in the infected areas. The role of bone marrow scan with ^99m^Tc-labeled sulfur colloid is to aid in delineating the patient's hematopoietic marrow distribution, thus acting as a comparator to the marrow uptake by besilesomab^4^. It is considered to be positive for infection when the findings from both NM procedures are dissimilar or spatially incongruent (uptake of besilesomab is higher than sulfur colloid). Here, the uptake for ^99m^Tc-besilesomab was shown to be similar to ^99m^Tc-sulfur colloid, hence negative for PMI. However, it is interesting to mention that the active compound found in besilesomab may trigger the formation of human anti-mouse antibodies (HAMA), which could alter the biodistribution of subsequent radiotracer injections and result in an allergic response^4^. To prevent such events, the determination of HAMA levels is warranted before anti-granulocyte studies, and the re-administration to HAMA-positive patients is inadvisable.

Our confidence was increased even further with the use of hybrid SPECT/CT. It provided additional anatomic information during the ^99m^Tc-besilesomab scan and was able to exclude any suspected foci of uptake by showing correct localisation of marrow uptake. Although the latest recommendations for interpreting anti-granulocyte scintigraphy are strictly based on traditional planar images^2^, adding SPECT/CT for demanding cases such as PMI can be very beneficial as it increases the overall diagnostic accuracy.

The role of NM imaging shown here is clear, but crucial challenges remain in the interpretation of orthopaedic infections with megaprosthesis. In summary, we are able to exclude PMI by using besilesomab and sulfur colloid scintigraphy, together with hybrid SPECT/CT. The utility of this advanced imaging technique is pivotal as it prevents unnecessary interventions that could lead to permanent disability, a prolonged course of antibiotics, and an increased economic burden. Furthermore, removal of the endoprosthesis can be avoided, and a two-stage revision infected prosthesis surgery can be disabled. With hybrid imaging units such as SPECT/CT becoming more accessible, it is anticipated that nuclear medicine physicians, especially in Southeast Asia, will increase its utilisation for routine assessment of musculoskeletal infection.

## References

[1] Corona PS, Vicente M, Lalanza M, Amat C, Carrera L (2018). Use of modular megaprosthesis in managing chronic end-stage periprosthetic hip and knee infections: Is there an increase in relapse rate?. Eur J Orthop Surg Traumatol..

[2] Signore A, Sconfienza LM, Borens O, Glaudemans AWJM, Cassar-Pullicino V, Trampuz A (2019). Consensus document for the diagnosis of prosthetic joint infections: a joint paper by the EANM, EBJIS, and ESR (with ESCMID endorsement). Eur J Nucl Med Mol Imaging..

[3] Tande AJ, Patel R. (2014). Prosthetic joint infection. Clin Microbiol Rev..

[4] Seltzer A, Xiao R, Fernandez M, Hasija R (2019). Role of nuclear medicine imaging in evaluation of orthopedic infections, current concepts. J Clin Orthop Trauma..

[5] Wouthuyzen-Bakker M, Lora-Tamayo J, Senneville E, Scarbourough M, Ferry T, Uckay I (2018). Erysipelas or cellulitis with a prosthetic joint in situ. J Bone Jt Infect..

